# Metabolic control of systemic lupus erythematosus: convergence of genetic and environmental factors on mitochondrial dysfunction and mTOR reveal treatment targets in lupus

**DOI:** 10.1186/ar3969

**Published:** 2012-09-27

**Authors:** A Perl

**Affiliations:** 1 State University of New York, Upstate Medical University, College of Medicine, Syracuse, NY, USA

## 

Systemic lupus erythematosus (SLE) is characterized by the dysfunction of T cells, B cells, and dendritic cells, the release of proinflammatory nuclear materials from necrotic cells and the formation of antinuclear antibodies (ANA) and immune complexes of ANA with DNA, RNA, and nuclear proteins [1]. Oxidative stress and inflammation lead to parenchymal and vascular tissue damage, the latter resulting in accelerated atherosclerosis that is a major cause of mortality in SLE. Activation of the mammalian target of rapamycin (mTOR) has recently emerged as a key factor in abnormal activation of T cells and B cells in SLE [2]. In T cells, increased production of nitric oxide and mitochondrial hyperpolarization (MHP) were identified as metabolic checkpoints upstream of mTOR activation. mTOR controls the expression T-cell receptor-associated signaling proteins CD4 and CD3ζ through increased expression of the endosome recycling regulator Rab5 and HRES-1/Rab4 genes [3], enhances Ca^2+ ^fluxing and skews the expression of tyrosine kinases both in T cells and B cells, and blocks the expression of Foxp3 and the generation of regulatory T cells [4]. MHP, increased activity of mTOR, Rab GTPases, and Syk kinases, and enhanced Ca^2+ ^flux have emerged as common T-cell and B-cell biomarkers and targets for treatment in SLE [5]. While inactivation and depletion of B cells have shown success in both animal models and patients, blockade of oxidative stress [6], mTOR [7], tyrosine kinases and T-cell-B-cell interaction are also being evaluated as targets for treatment in SLE.
